# Transcanalicular diode laser assisted dacryocystorhinostomy: A breakthrough in the treatment of acquired nasolacrimal duct obstruction

**DOI:** 10.12669/pjms.36.4.1906

**Published:** 2020

**Authors:** Muhammad Awais, Syed Abid Hassan Naqvi, Amjad Akram, Muhammad Shahid

**Affiliations:** 1 Dr. Muhammad Awais. FCPS (Ophth), Armed Forces Institute of Ophthalmology (AFIO) Rawalpindi, Pakistan; 2 Dr. Syed Abid HassanNaqvi. FCPS (Ophth), FRCS., Armed Forces Institute of Ophthalmology (AFIO) Rawalpindi, Pakistan; 3 Dr. Amjad Akram. FCPS. FRCS (Glasgow). FRCS (Ed.), Armed Forces Institute of Ophthalmology (AFIO) Rawalpindi, Pakistan; 4 Dr. Muhammad Shahid. FCPS (Ophth), Armed Forces Institute of Ophthalmology (AFIO) Rawalpindi, Pakistan

**Keywords:** Epiphora, Transcanalicular Dacryocystorhinostomy, Diode laser

## Abstract

**Objective:**

To evaluate the success rate of Transcanalicular Diode laser assisted Dacryocystorhinostomy (TC-DCR) in cases of acquired nasolacrimal duct obstruction (NLDO).

**Methods:**

This Quasi-Experimental study was conducted in Armed Forces Institute of Ophthalmology (AFIO) Rawalpindi, from July 2018 to April 2019. In this study, 73 patients of chronic dacryocystitis secondary to acquired NLDO were treated by TC-DCR under general anaesthesia. Follow up examination was done after one day, one week and three months. Silicone tubes were removed three months after surgery and syringing of lacrimal system done to confirm patency of lacrimal passages. Success of the procedure was documented as absence of epiphora and patent nasolacrimal duct on syringing.

**Results:**

Seventy three patients (males 27; females 46) were included in this study. Mean age of these patients was 51.6+21 years. On completion of this study which was three months after surgery, we found subjective improvement (absence of epiphora) in 86.3% patients and objective improvement (successful irrigation of lacrimal passages) in 93.2% patients.

**Conclusion:**

TC- DCR is a minimally invasive technique of doing Dacryocystorhinostomy and imparts more than 90% success rate. It has additional advantages of good cosmetic results, low complication rate and short surgery and convalescence time.

## INTRODUCTION

Chronic dacryocystitis is chronic inflammation of lacrimal sac and its most common cause is occlusion of nasolacrimal duct.^1^ If untreated, it may cause recurrent conjunctival inflammation^2^, dacryocoele, lacrimal sac fistula and even orbital cellulitis. Treatment of choice for chronic dacryocystitis is dacryocystorhinostomy (DCR) in which a permanent passage is created between lacrimal sac and nasal cavity. DCR through external approach, due to high success rate (>90%) remained the gold standard technique for decades.^3^ Caldwell in 1893 described an alternative to DCR by doing it via endonasal approach with the help of nasal endoscope.^3^ Later on, because of the innovation of higher resolution fiber optic nasal endoscopes, endoscopic DCR started becoming popular. The recent advancement in DCR technique is doing it via transcanalicular approach, in which osteotomy is created with the help of diode laser.^4^ Diode laser assisted TC-DCR has some additive advantages as it can be done under local anesthesia, causes precise cutting and removal of tissue by ablation, is bloodless, less time-consuming, leaves no external scars and can be repeated.^5^,^6^ It can also be used to treat failed Ex-DCR.

Diode laser-assisted TC-DCR has become the topic of several current papers internationally. But the literature search showed that no study has, so far, been conducted on Pakistani population, in this regard. The rationale of doing this study was to determine and publish the success rate of diode laser assisted TC-DCR in Pakistani patients of primary acquired NLDO.

## METHODS

This Quasi-experimental study comprises 73 Patients who underwent diode laser assisted TC-DCR between July 2018 and April 2019. Approval for the study was granted by the Institutional Review Board. Patients of chronic dacryocystitis secondary to primary acquired NLDO were included in the study. The exclusion criteria included acute dacryocystitis, common canalicular obstruction, severe facial trauma causing disfigurement and nasal polyp. Patients reporting in the OPD of AFIO, with the complaint of epiphora were examined and those with the problems of puntal stenosis, allergic conjunctivitis, blepharitis, dry eyes, corneal or conjunctival infections were excluded. After screening, these patients underwent syringing and lacrimal irrigation and patients with blocked nasolacrimal duct were selected for the surgical procedure. Written informed consent was obtained from these patients. Pre-operative anaesthesia assessment of these patients was done by a consultant anaesthetist. Endoscopic nasal examination of these paients was done to exclude deviated nasal septum or nasal polyps. For TC-DCR procedure, we used KLS Martin DiomaX machine. Our preferred settings are three Watt power in pulse mode, pulse duration two sec, pause between pulses 0.4 sec, and so average power is 2.5 watt. The laser was delivered through 600 um flexible fibre optic probes.

We performed all the cases under general anaesthesia. Nasal packing was done with neuro patties (ribbon gauze) soaked in a solution containing lignocaine and adrenaline in 1: 10,000 ratio. A solution of epinephrine 1: 100,000 was infiltrated into nasal mucosa of lateral nasal wall so that per operative bleeding may be minimized. Both upper and lower puncta were dilated and a bowman lacrimal probe was passed to feel hard stop. Fiber optic laser probe was passed through one punctum into the lacrimal sac. Zero degree nasal video endoscope was introduced into the respective nasal cavity and the red beam translucency of laser probe was seen in the nasal cavity. The site of osteotomy in this procedure is just anterior and inferior to the attachment of middle nasal concha to the lateral nasal wall. Tip of the Diode laser probe was in direct contact with the medial wall of lacrimal sac. Then the laser was fired repeatedly which ablated lacrimal sac tissue, lacrimal bone and nasal mucosa and thus created an opening. This osteotomy was then enlarged to about five mm by applying adjacent burns. Bicanalicular silicone tubes were passed through the punta and retrieved through nostril and tied in the nasal cavity. Post operatively, nasal cavity was packed using ribbon gauze soaked in a solution of two percent lignocaine with adrenaline to minimize bleeding from nasal mucosa. This nasal packing was removed next morning. Patients were prescribed Tab Amoxycillin-Clavulanic acid, one gram 12 hourly and Tab Paracetamol 500 mg 12 hourly for three days. Tobramycin plus dexamethasone eye drops were prescribed four times daily for one month. Oxymetazoline nasal spray was prescribed to use, eight hourly, daily for two weeks. Follow-up visits were scheduled on one day, one week and then three months after surgery. After three months of surgery, silicone tubes were removed and all the patients underwent lacrimal syringing to confirm patency of the lacrimal system. Success of the procedure was determined as absence of epiphora/discharge and patent nasolacrimal duct on irrigation.

Variables like age, gender, post- operative epiphora and post-operative regurgitation were noted. Statistical package for social sciences (SPSS 25) was used for statistical analysis.

## RESULTS

Seventy three patients underwent diode laser assisted TC-DCR. Out of these, 27 (37%) were males and 46 (63%) females. Mean age of these patients was 51.6 + 21 years. Thirty nine cases were of right NLDO whereas 33 cases were of left NLDO. One patient underwent bilateral DCR. We divided parameters of success (lacrimal patency) into subjective (absence of epiphora) and objective (successful irrigation of lacrimal passages) ones. We found absence of epiphora in 63 (86.3%) patients whereas 10 (13.7%) patients complained of persistent watery discharge three months after surgery. Post-operative epiphora occurred in eight (80%) males and two (20%) females (Table-I); p-value is 0.002 which is significant. On irrigation of lacrimal passage, we found objective improvement in 68 (93.2%) patients. In these patients, saline jet injected through punctum reached pharynx. Saline jet regurgitated back only in five (6.8%) patients. Post-operative regurgitation occurred in three (60%) males and two (40%) females (Table-I); p-value is 0.269 which is not significant. Anatomical success rate in our patients after transcanalicular diode laser assisted DCR was 93.2%.

**Table-I:** Crosstabulation between gender and post-operative epiphora and post- op regurgitation.

Gender	Post Op Epiphora		Post Op Regurgitation	

	Yes	No	Yes	No
Male	8 (80.0%)	19 (30.2%)	3 (60.0%)	24 (35.3%)
Female	2 (20.0%)	44 (69.8%)	2 (40.0%)	44 (64.7%)

## DISCUSSION

The modern era aims and strives for least traumatic surgical procedures which impart good cosmetic results, and early post-operative recovery. Ophthalmology is no stranger to this. External Dacryocystorhinostomy (Ex DCR) was previously considered the gold standard technique for the treatment of chronic dacryocystitis.^7^ Though it yielded good anatomical and functional success, the issues of cutaneous scar, prolonged procedure time and per operative bleeding could not be addressed. Dacryocystorhinostomy via endonasal approach was also not devoid of the issues of per-operative bleeding and long learning curve.

Transcanalicular laser assisted DCR (TC-DCR) is a minimally invasive alternative to Ex DCR.^8^,^9^ The obvious advantages of avoiding an external incision^10^ and fast post-operative recovery made it attractive to many patients.^6^ Short learning curve, less procedure time^11^, minimal per-operative bleeding during TC-DCR is gaining attention of ophthalmologists. This procedure can be used for revision of failed Ex DCR. It can be performed under local anaesthesia and can protect the patients from the risks of general anaesthesia.^12^ Lasers with several wavelengths have been used to perform osteotomy; some of these are Holmium:Yttrium-Aluminum-Garnet (Ho:YAG) laser, Neodymium:YAG (Nd:YAG) laser, Erbium:YAG (Er:YAG) laser and Diode laser.^11^ Diode laser has some peculiar advantages over other laser types. For example, this is a contact laser and the energy emitted is confined to the tip of laser probe. Secondly presence of an insulating sleeve around the probe minimizes collateral heating. It also achieves effective tissue dissection, precise cutting and removal of tissue by ablation resulting in minimal haemorrhage and improved intraoperative view.^1^

In this prospective study, we performed TC-DCR on 73 patients out of which 27 (37%) were males and 46 (63%) were females. Lacrimal sac problems are found more common in females.^13^,^14^ In a study conducted by Al-Asaadi SZ, the male patients were 31.35% while the females were 68.75%.^15^ This is close to our results. Lacrimal passageway was open in 93.2% of our operated patients three months after surgery. Our results are close to that of Ex DCR (90-95%).^16^ This high success rate is attributed to few factors. The operating surgeon was quite familiar with the nasal anatomy and made adequate sized osteotomy by visualizing through nasal endoscope. The use of silicone tubes also enhanced our success rate. Because of Diode laser, minimal trauma occurred to the surrounding tissue leading to minimal post- operative inflammation and scarring at osteotomy site which also reduced chances of procedure failure. However, results of TC- DCR are quite variable in published literature. Brigita Drnovsek-Olup reported success rate of 83.3%.^11^ Salah Zuhair Al-Asadi reported success rate of 93.75% after performing TC-DCR with silicone intubation.^15^ Success rate of 90% is reported by Goel R et al.^1^ A functional success rate of 74-85% is also reported at one year follow-up.^6^,^11^,^17^-^21^ But contrary to above, Kaynak et al. reported that the success rate of TC-DCR drops to 60% within two years.^22^ The differences in success rates are probably because of use of different laser settings by different people, preference of silicone intubation, local vs general anaesthesia and use of adjunctive antimetabolites like Mitomycin C at osteotomy site. To improve success rate, most important step is the correct formation of an adequate sized osteotomy at the optimal location.^23^,^24^ It is better to aim for as big an osteotomy as possible while at the same time avoiding unnecessary tissue damage. A limitation of this study was its shorter follow-up period. Concerns are about long-term success of TC-DCR. Hence, further studies need to be conducted with long-term follow-up.

## CONCLUSION

Transcanalicular laser-assisted DCR provides good results and is a viable option before proceeding to more invasive lacrimal surgery in cases of nasolacrimal duct obstruction. Because of low complication rates and short convalescence and surgery time, patient satisfaction is high. While at the same time, functional success rates range closely behind those of external DCR.

### Authors’ contribution

**MA:** Collected data and did manuscript writing.

**SAHN:** Performed surgical procedure on selected patients and did proof reading of manuscript.

**AA:** Did analysis of data and contributed in manuscript writing.

**MS:** Contributed in the surgery of selected patients and contributed in manuscript writing.
